# Ketamine interactions with gut-microbiota in rats: relevance to its antidepressant and anti-inflammatory properties

**DOI:** 10.1186/s12866-018-1373-7

**Published:** 2018-12-22

**Authors:** Bruk Getachew, Joseph I. Aubee, Richard S. Schottenfeld, Antonei B. Csoka, Karl M. Thompson, Yousef Tizabi

**Affiliations:** 1 Department of Pharmacology, Howard University College Medicine, 520 W Street NW, Washington, DC 20059 USA; 2 Department of Microbiology, Howard University College Medicine, Washington, DC 20059 USA; 3 Department of Psychiatry and Behavioral Sciences, Howard University College Medicine, Washington, DC 20059 USA; 4 Department of Anatomy, Howard University College Medicine, Washington, DC 20059 USA

**Keywords:** NMDA receptor, *Lactobacillus*, *Turicibacter*, *Ruminococcus*, *Sarcina*, *Mucispirillum*, 16S rRNA gene, Metagenomics, Dysbiosis, Inflammatory diseases, Depression

## Abstract

**Background:**

Appreciable evidence suggest that dysbiosis in microbiota, reflected in gut microbial imbalance plays a key role in the pathogenesis of neuropsychiatric disorders including depression and inflammatory diseases. Recently, the antidepressant properties of ketamine have gained prominence due to its fast and long lasting effects. Additional uses for ketamine in inflammatory disorders such as irritable bowel syndrome have been suggested. However, ketamine’s exact mechanism of action and potential effects on microbiome is not known. Here, we examined the effects of low dose ketamine, known to induce antidepressant effects, on stool microbiome profile in adult male Wistar rats. Animals (5/group) were injected intraperitoneally with ketamine (2.5 mg/kg) or saline, daily for 7 days and sacrificed on day 8 when intestinal stools were collected and stored at − 80 °C. DNA was extracted from the samples and the 16 S rRNA gene-based microbiota analysis was performed using 16S Metagenomics application.

**Results:**

At genus–level, ketamine strikingly amplified *Lactobacillus*, *Turicibacter* and *Sarcina* by 3.3, 26 and 42 fold, respectively. Conversely, opportunistic pathogens *Mucispirillum* and *Ruminococcus* were reduced by approximately 2.6 and 26 fold, respectively, in ketamine group. Low levels of *Lactobacillus* and *Turicibacter* are associated with various disorders including depression and administration of certain species of *Lactobacillus* ameliorates depressive-like behavior in animal models. Hence, some of the antidepressant effects of ketamine might be mediated through its interaction with these gut bacteria. Additionally, high level of *Ruminococcus* is positively associated with the severity of irritable bowel syndrome (IBS), and some species of *Mucispirillum* have been associated with intestinal inflammation. Indirect evidence of anti-inflammatory role of *Sarcina* has been documented. Hence, some of the anti-inflammatory effects of ketamine and its usefulness in specific inflammatory diseases including IBS may be mediated through its interaction with these latter bacteria.

**Conclusion:**

Our data suggest that at least some of the antidepressant and anti-inflammatory effects of daily ketamine treatment for 7 days may be mediated via its interaction with specific gut bacteria. These findings further validate the usefulness of microbiome as a target for therapeutic intervention and call for more detailed investigation of microbiome interaction with central mediators of mood and/or inflammatory disorders.

## Background

Converging evidence suggests that the brain and the gut microbiota are in bidirectional communication with each other and also with inflammatory processes [1–5]. Thus, on the one hand, dysbiosis, an imbalance in the microbiota community, may occur in depression and chronic stress due to altered brain signaling to the gut [6]. On the other hand, altered gut microbial signaling to the brain may result in brain alterations [7]. Clinical evidence supporting gut microbiota-brain-inflammatory processes interaction, specifically in relation to mood dysregulation such as in major depressive disorder (MDD) include: 1. depressive symptoms are often co-morbid with gastrointestinal (GI) disorders such as metabolic syndrome, inflammatory bowel disease, and irritable bowel syndrome (IBS) [8–12]; 2. this comorbidity presents increased risk for disease progression and poorer outcome, and treatment of one condition can reverse the risk for the other [13–17]; 3. some classes of antibiotics have been shown to have antidepressant effects [18–22]. Conversely, some antidepressants may also possess antimicrobial properties [23, 24]. Moreover, germ-free animals show increased depressive-like behavior that can be reversed by administering a single bacterium such as *Bifidobacterium infantis* [25, 26]. Similarly, significant depletion of the gut microbiota with selective antibiotics can result in depressive-like phenotype [27]. Fecal microbiota transplantation from depressed patients to microbiota-depleted rats induces behavioral and physiological characteristic of depression (i.e., anhedonia and altered tryptophan metabolism) in these rats [4, 28]. Conversely, probiotics consumption can increase plasma levels of tryptophan and reduce levels of the pro-inflammatory cytokines such as interleukin-1-beta (IL-1β), interleukin-6 (IL-6) and tumor necrosis factor-alpha (TNFα). Interestingly, reduction of these pro-inflammatory cytokines can result in abatement of depressive-like behavior [29]. Overall, these data suggest involvement of microbiome in pathogenesis of depressive behavior and possibly the effectiveness of antidepressants.

Off-label use of ketamine for depression is becoming prominent due to its prompt and sustained antidepressant effects. Indeed, (S)-ketamine (esketamine) is in approval process by FDA as a fast-acting antidepressant with particular application in treatment-resistant depression and suicidal ideation [30]. The purported effects of acute ketamine include inhibition of NMDA (N-methyl-d-aspartate) receptors and activation of AMPA (α-amino-3-hydroxy-5-methyl-4-isoxazolepropionic acid) receptors as well as molecular signaling of mTOR (the mammalian target of rapamycin), which result in enhancement of hippocampal brain-derived neurotrophic factor (BDNF) and increased synaptogenesis [31–35]. The sustained effectiveness of acute ketamine is likely mediated by additional mechanisms as increased BDNF levels are not maintained beyond 24 h after ketamine administration [36]. In this regard, interaction of acute ketamine with gut microbiota has recently been reported [37, 38]. Moreover, it was demonstrated that gut microbiome is capable of modulating central BDNF [39, 40]. However, to our knowledge no study has examined the effects of chronic ketamine on gut microbiota.

In addition to its well established antidepressant effects, ketamine has also been advocated for use in inflammatory diseases such as ulcerative colitis [41, 42]. However, here also, no studies on interaction between ketamine and gut microbiota implicated in inflammatory diseases has been carried out. Thus, this study was conducted to determine the effects of chronic ketamine on gut microbiota, especially those implicated in mood regulation and/or inflammatory responses. Our hypothesis was that ketamine would promote microbiota implicated in mood elevation and suppress microbiota implicated in inflammatory diseases.

## Methods

### Animals

Age matched, approximately 3 months old adult male Wistar rats (Evigo, USA) were housed 2–3 per cage in standard polypropylene shoebox cages (42 × 20.5 × 20 cm) on hardwood chip bedding (alpha-dry) in a designated room. Throughout the experiment, animals had access to food (Harlan Tek Lab) and water ad libitum. The room was maintained at 24–26 °C at 51–66% relative humidity, on a 12-h light/dark cycle (lights on at 7 am).

In order to acclimate the subjects to the housing conditions, animals arrived at least one week prior to initiation of any experiment. During this period, they were gentled once daily in order to minimize any stress effects that might result from routine handling.

### Drugs

A ready-made preparation KETAHESIA® inj. Sol. purchased from Henry Schein (Dublin, OH) (100 mg/ml), was diluted with saline to obtain desired concentration of 2.5 mg/ml.

### Experimental design

Following one week of acclimation, the animals were randomly divided into two groups, control and experimental (*n* = 5 each) and were housed in separate cages. Animals belonging to the same group were also randomly selected and housed together (2–3 animals/cage). This housing method assured that the animals in both groups were exposed to identical environment and that there would not be any cross-contaminatin between the treated vs the control group. The number of animals used in each group was based on behavioral observations seen using similar number of animals.

Control group was injected daily (around noon) with saline, whereas the experimental group received ketamine (2.5 mg/kg). All injections were done intraperitoneally (i.p.) and were carried out for 7 consecutive days. The volume of injection was 1 ml/kg. The low dose of ketamine used in this study was based on our previous study, where clearly an antidepressant effect of such a dose and duration was observed [32, 43]

### Sample collection

On day 8, approximately 24 h after the last ketamine or saline injection, the animals were sacrificed by decapitation, alternating between the groups as described previously [44]. Colons containing stools were collected, quick-frozen on dry ice and stored at − 80 °C. This method of rapid–freezing is considered best-practice for preserving stool DNA samples [45].

### Stool DNA extraction

Total DNA was isolated from stool samples using Norgen’s Stool DNA Isolation Kit and the Precellys Dual-24 Homogenizer (Bertin Technologies). Purification was based on spin column chromatography using resin as the separation matrix. Briefly, 200 mg stool samples were bead-homogenized after adding 1 mL of Lysis Buffer L. One hundred μL of lysis additive was added and vortexed, followed by centrifugation at 20,000 x g for 5 min. The clear supernatant was transferred (600 μL) to a DNAase-free microcetrifuge tube. Next, the samples were centrifuged and 100 μL of Binding Buffer I was added to the clean supernatant and incubated on ice for 10 min. Equal amounts of 70% ethanol were then added to the clean supernatant from Binding Buffer I lysate after centrifugation. The protocol was then followed for complete DNA isolation. The purified DNA was quantified and analyzed for purity using the NanoDrop™ 2000 Spectrophotometer (NanoDrop Technologies, Wilmington, DE). Twenty μL of purified DNA was then quick-frozen on dry ice and shipped to Norgen Biotek (Thorold, ON, Canada) for 16S rRNA gene analysis.

### 16S rRNA gene sequencing and analysis

Briefly, the V3-V4 hypervariable region of the bacterial 16S rRNA gene was amplified from 12.5 ng of stool DNA. The amplicons were then cleaned, sequenced according to the Illumina MiSeq 16S Metagenomic Sequencing Library Preparation protocol [46]. The final library was paired-end sequenced at 2 × 300 bp using a MiSeq Reagent Kit v3 on the Illumina MiSeq platform. For bioinformatic analysis, the sequencing data was analyzed using the Illumina 16S metagenomics app (Illumina 16S Metagenimics Pippeline (v1.0.1) [47], which performs taxonomic classification of 16S rRNA targeted amplicons using an Illumina-curated version of the GeenGenes taxonomic database. The app provides interactive visualization and raw classification output for per-sample and aggregate analyses. Classification was performed using the Illumina 16 S Metagenomics workflow, which is also available in the MiSeq Reporter software. The algorithm uses a high-performance implementation of the Ribosomal Database Project (RDP) Classifier described in Wang et al., 2007 [48].

### Statistical analysis

Since comparison was performed between two groups with equal variance (ketamine- and saline-treated animals), Student T-test was applied for detecting significant differences in specific measured parameters. The cut-off for statistical significance was *p* < 0.05, two-tailed

## Results

### Diversity and richness

**Fig. 1:**
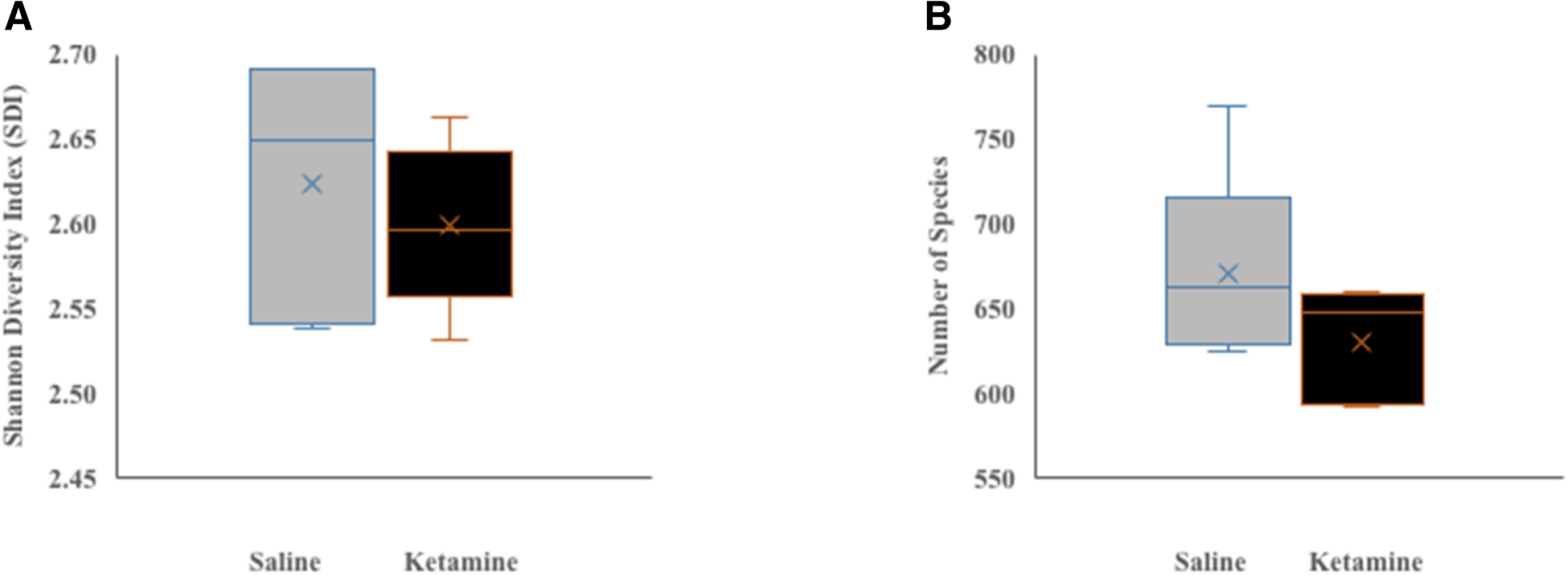
Effects of 7 day ketamine (2.5 mg/kg i.p.) on gut bacterial species diversity (**a**) and species richness (**b**). Box-and-Whisker represent values of Shannon Diversity Index (*n* = 5) for (**a**) and species number (**b**). Ketamine did not have any significant effect on species diversity (*P* = 0.99) or species richness (*P* = 0.22). The ‘x’ designation in the middle of the bar represents mean value for each group, horizontal line across the bars indicate median values. The minimum and maximum quartile values are represented by a horizontal line in the bottom and top of the bars

Figure 1 depicts the effects of chronic low dose ketamine on gut bacterial diversity (A) and richness (B). A total of 1121 different bacterial species were identified in both saline and ketamine groups. Overall, there was no significant difference in either diversity (Fig. 1a) as estimated by the Shannon Diversity Index (SDI) (saline control vs. ketamine; 2.62 vs. 2.60, *P* = 0.99) or species richness (Fig. 1b) as measured by mean species number. Although a total of 1121species were identified, only less than 700 (saline control vs. ketamine; 630 vs. 670, *P* = 0.22) were considered qualified (i.e., made the cut off at 0.01% abundance).

### Taxa-level distribution

**Fig. 2:**
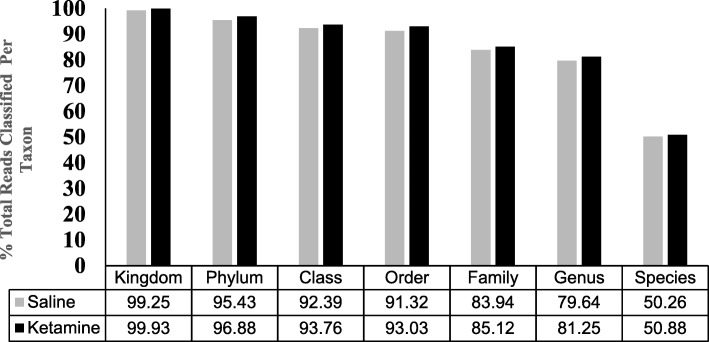
Effects of 7 day ketamine (2.5 mg/kg i.p.) on gut bacterial distribution of taxa. Values are percent total reads/taxa/group (*n* = 5). There were no significant differences in the % reads in taxon between saline and ketamine group. Note: the samples from each group were pooled and hence overall there were two groups to be compared (control vs treated)

There were a total number of 29 Phyla, 56 classes, 106 orders, 234 families, 600 genera and 1121 species identified in the two groups. There were no differences between the saline and ketamine group in percent reads, i.e., percentage of identified sequences belonging to each taxon (Fig. 2).

### Phylum-level effects

**Fig. 3:**
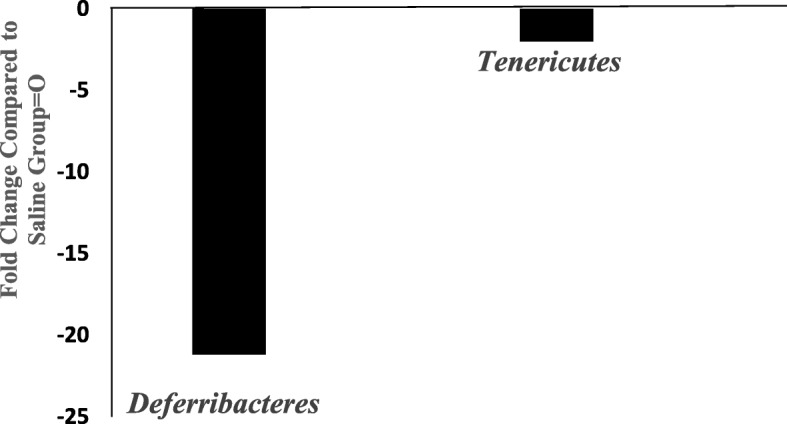
Effects of 7 day ketamine (2.5 mg/kg i.p.) on fold change in abundance of gut bacterial phylum. Values are the fold change of mean compared to saline-control group (*n* = 5). Ketamine substantially decreased *Deferribacteres* and *Tenericutes* compared to saline group by 22 and 2.3 fold, respectively. Note: the samples from each group were pooled and hence overall there were two groups to be compared (control vs treated)

Ketamine significantly reduced abundances of two phyla, *Deferibacteres* and *Tenericutes* (Fig. 3). Overall there were 29 different phyla identified in the two groups. *Deferibacteres* and *Tenericutes* are low-abundance phyla accounting for less than 2% of the total phyla reads. Ketamine selectively reduced *Deferibacteres* and *Tenericutes* by approximately 22 and 2 fold, respectively, compared to saline control group.

### Class-Level Effects

**Fig. 4:**
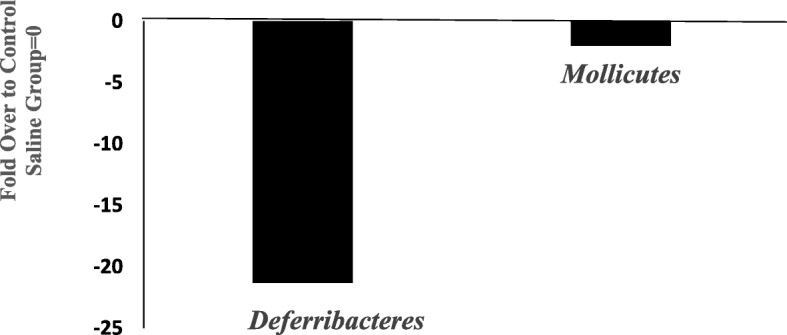
Effects of 7 day ketamine (2.5 mg/kg i.p.) on fold change in abundance of gut bacterial class. Values are the fold change over saline control group mean (*n* = 5). Ketamine significantly decreased *Deferribacteres* and *Mollicutes* compared to saline group by 22 and 2 fold, respectively. Note: the samples from each group were pooled and hence overall there were two groups to be compared (control vs treated)

Ketamine significantly reduced the levels of two classes, *Deferrribacteres* and *Mollicutes* by 22 and 2 fold, respectively, compared to the saline group (Fig. 4). There were a total of 56 different classes identified between the two groups.

### Order-level effects

**Fig. 5:**
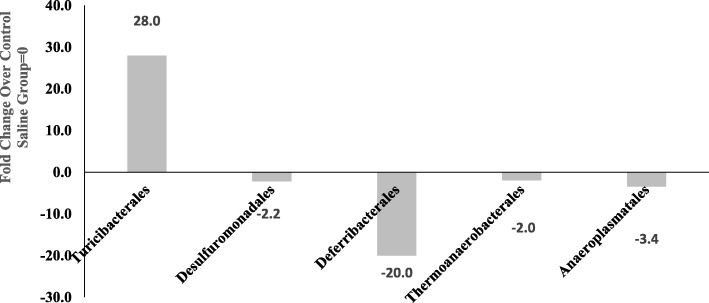
Effects of 7 day ketamine (2.5 mg/kg i.p.) on fold change in abundance of gut bacterial order. Values are the fold change over saline control group mean (*n* = 5). Ketamine significantly increased *Turicibacterales* by 28 fold and decreased *Desulfuromonadales*, *Deferribacterales*, *Theromonaerobacterales* and *Anaeroplasmatales* by 2.2 and 3.4 fold, respectively, compared to saline group. There were a total of 106 different orders. Numbers on the columns refer to the fold-change. Note: the samples from each group were pooled and hence overall there were two groups to be compared (control vs treated)

Ketamine significantly increased the abundance of *Turicibacterales* order by 28 fold, and reduced the abundance of four orders: *Desulfuromonadales*, *Deferribacterales*, *Theromonaerobacterales* and *Anaeroplasmatales* by 2.2, 20, 2 and 3.4 fold, respectively, compared to saline control group (Fig. 5). There were a total of 106 different classes identified in both groups.

### Family-level effects

**Fig. 6:**
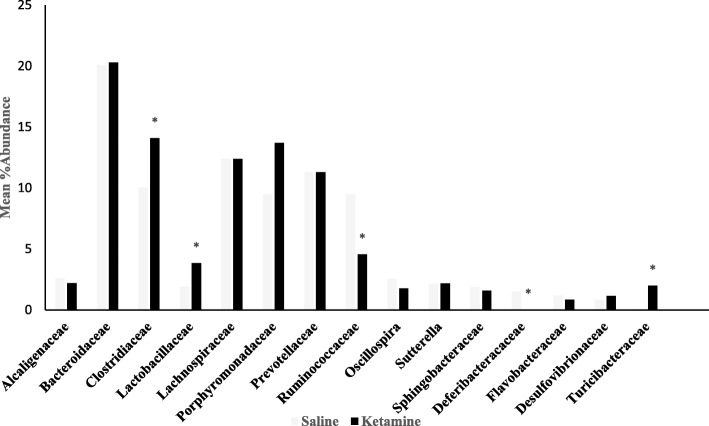
Effects of 7 day ketamine (2.5 mg/kg i.p.) on percent abundance of gut bacterial family. Values are mean % abundance of group mean (*n* = 5). Ketamine significantly enriched abundance of genera *Lactobacillaceae*, *Turicibateraceae*, *Clostridiaceae* by 1.5, 98 and 89 fold respectively. Conversely, ketamine reduced abundance of *Ruminococcaceae* and *Deferribacteraceae* by 2.3 and 26.3 folds respectively compared to saline group. * indicate significant changes compared to saline. There were a total of 234 families

Ketamine significantly enriched *Tuberibacteraceae* by 98 fold, *Clostridiaceae* by 89 fold and *Lactobacillaceae* by1.5 fold, whereas *Deferrribacteraceae* and *Ruminococcaceae* were reduced by approximately 26 and 2.3 fold, respectively, compared to the saline control group at the family-level (Fig. 6). There were a total of 234 different families. It should be noted that for analysis at the family level, the samples were pooled and hence overall there were two gorups to be compared (control vs treated). Since a statistical analysis could not be perforemed in such cases, we used a conservative cutoff point of a minimum of 1.5 fold difference between the gorups, which could imply important changes.

### Genus-level effects

**Fig. 7:**
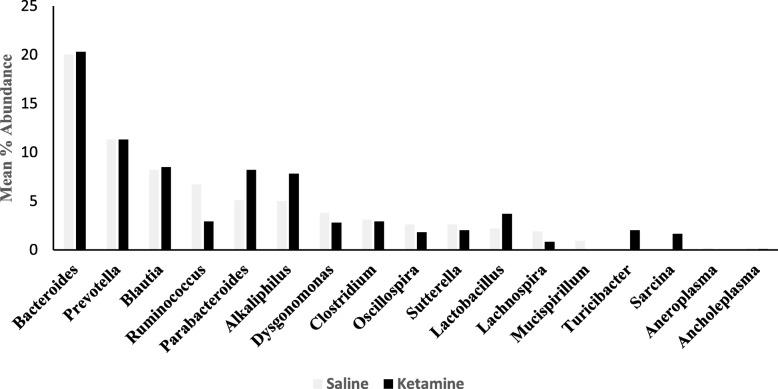
Effects of 7 day ketamine (2.5 mg/kg i.p.) on percent abundance of gut bacteria genus. Values are mean % abundances of group mean (*n* = 5). Ketamine significantly enriched abundance of genera *Sarcina*, *Turicibater*, *Lactobacillus* by 42, 20 and 2 fold, respectively. Conversely, ketamine decreased levels of *Mucispirillum* and *Ruminococcaceae* by 26.3 and 2.3 fold, respectively. All comparisons are with respect to saline group. There were a total of 600 different genera

Ketamine significantly enriched abundances of genera *Sarcina*, *Turicibater*, *Lactobacillus* by 42, 20 and 2 fold respectively, Whereas, levels of *Mucispirillum* and *Ruminococcaceae* were decreased by 26.3 and 2.3 fold, respectively, compared to saline control group (Fig. 7). There were a total of 600 different genera. It should be noted that for analysis at the genus level, the samples were pooled and hence overall there were two gorups to be compared (control vs treated). Since a statistical analysis could not be perforemed in such cases, we used a conservative cutoff point of a minimum of 1.5 fold difference between the gorups, which could imply important changes.

## Discussion

Our data indicates drastic effects of chronic low dose ketamine on gut microbial ecology, ranging from 2 to 42 fold changes in specific genera. For example, *Lactobacillus* and *Turicibacter* were increased by approximately 2 and 20 fold, respectively, whereas *Mucispirillum* and *Ruminococcus* were reduced by approximately 26 and 2.3 fold, respectively. Since low levels of *Lactobacillus* and *Turicibacter* are associated with various disorders including depression [49–51], it might be suggested that these gut microbiomes may play a role in ketamine’s antidepressant properties. Further support for this contention is provided by findings that administration of *Lactobacillus* ameliorates depressive-like behavior in animal models [49, 52–54]. As mentioned earlier, antidepressant effects of low dose ketamine have been amply supported in both preclinical as well as clinical studies [30–35].

Conversely, elevated levels of *Mucispirillum* and *Ruminococcus* are associated with inflammatory processes [55–59]. Indeed, high levels of *Ruminococcus* has been shown to be positively associated with the severity of irritable bowel disease and some species of *Mucispirillum* may cause intestinal inflammation [58–63]. Hence, reduction of these microorganisms by ketamine, may suggest an additional novel mechanism for ketamine’s anti-inflammatory effects. On the other hand, since low levels of *Sarcina* has been implicated in inflammatory processes [64], its 42-fold magnification by ketamine may also be a contributory factor to ketamine’s anti-inflammatory properties. Here also, as noted earlier, anti-inflammatory effects of ketamine are well documented [41, 42].

In addition, changes in low-abundance groups such as *Lactobaccilus*, *Sarcina* and *Turicibacter* can markedly influence the gut ecosystem [65, 66]. For example, these groups contain species that can degrade complex polysaccharides to short chain fatty acids such as butyrate [67, 68]. Changes in these “butyrogenic” bacteria may in turn influence metabolic, inflammatory bowel or neurological/neuropsychiatric disorders [69]. Indeed, butyrate, which can be used as a source of energy by the host, confers many benefits including anti-inflammatory effects [70]. Butyrate can also inhibit histone deacetylase (HDAC), resulting in increased levels of histone acetylation, thereby affecting gene expression [71]. Curiously, modest antidepressant-like effects of sodium butyrate, a weak and highly non-specific inhibitor of class I and class II HDACs, have been reported [72, 73]. Furthermore, by lowering colonic pH, butyrate may confer an added advantage for probiotic microbiota such as *Turicibacter*, which thrive in lower pH [68]. Curiously, Westernized diet implicated in many disorders including metabolic syndrome, is linked to lower levels of *Lactobacillus* and *Sarcina*, but higher levels of *Ruminococcus* [64, 74]. *Mucispirillum* is a gram-negative anaerobic bacterium that can constantly generate lipopolysaccharide (LPS) as an integral component of its outer cell membrane [59, 75]. This and other opportunistic bacteria flourish in gut environment causing inflammation and dysbiosis [55, 57, 58]. To this effect, an association between an increase in *Ruminococcus* level and diverticulitis, total hip arthroplasty, IBS severity and exercise-induced stress have been reported [56, 59, 76, 78]. Interestingly, exercise-related stress may have similar effects on microbiome. Thus, it was shown that forced exercise reduces levels of *Turicibacter*, and increases cecal *Ruminococcus* leading to intestinal inflammation, whereas voluntary wheel running for 6 weeks attenuates symptoms in a colitis mouse model [76].

A recent study by Qu et al. [37] indicates that acute (R)-ketamine significantly attenuated the increased levels of *Ruminococcaceae* (a family-taxa of *Ruminococcus*), in susceptible mice after chronic social defeat. Ketamine’s lowering of *Ruminococcaceae* and reversal of the behavioral deficits, including depressive-like behavior induced by social defeat, suggest that *Ruminococcaceae* may play a role in stress-induced depressive behavior [37]. Our results also suggest that antidepressant effects of chronic ketamine might be mediated through reduction of *Ruminococcus* in the gut. On the other hand, ketamine’s elevation of gut genera *Lactobacillus, Sarcina* and *Turicibacte*r reported here, suggests that reestablishment of gut equilibrium by chronic ketamine might be a contributory factor to its anti-inflammatory effects. This contention is further strengthened by findings that ameliorative effects of *Turicibacter* are correlated with increased intestinal butyric acid [50]. Thus, taken together, it might be suggested that chronic ketamine can ameliorate dysbiotic brought about by diet, stressful exercise or inflammation via its interaction with gut microbiome genera such as *Lactobacillus*, *Sarcina* and *Turicibacter*.

It is also of relevance to note that gut bacteria can influence the colonic mucus layer (mucin), a physical barrier that separates trillions of gut bacteria from the host [60, 62]. Some microbiota such as *Mucispirillum*, can increase gut penetrability leading to ‘leaky gut’ and reduce growth rate of the inner mucus layer [60–62]. Indeed, *Mucispirillum*, has a potential capacity to degrade mucin by actively destroying the microenvironment of the gut [60, 62], leading to leaky gut, which is considered a key contributor to the co-morbid condition of depression and intestinal disorders [77–79]. Moreover, *Mucispirillum* is positively associated with increases in plasma level of LPS, intestinal inflammation and severity of IBS [59]. *Mucispirillum* is also considered colitogenic and is used as a microbial marker in active colitis [58, 63]. Thus, some of the anti-inflammatory and possibly the antidepressant effects of ketamine might also be mediated through reduction of *Mucispirillum* and gut permeability. In this regard, future characterization of the relationship between *Mucispirillum* and affect is warranted.

Elucidation of the direct microbiome influence on neurobiological substrates of mood and on peripheral and central mediators of inflammatory processes may provide novel therapeutic targets in these disorders. It is noteworthy that many animal studies support the notion that central changes in cytokines and BDNF, both of which are directly linked to affective behavior, are influenced by gut microbiota. For example, oral administration of some antimicrobials to mice results in transient alteration of microbiota, increased hippocampal BDNF and antidepressant-like behavior [39]. However, i.p. administration of antimicrobials to mice or oral administration of antimicrobials to germ-free mice do not affect behavior, suggesting that changes in gut microbiomes are necessary to affect central BDNF levels and/or behavior [39]. Thus, inducing changes in the gut microbiota using probiotics, prebiotics or antimicrobial drugs are novel and promising targets in countering affective disorders [40]. In addition, as suggested earlier, manipulation of gut microbiome may also be a novel approach in combatting inflammatory disorders including colitis [80–82]. Therefore, further investigation of the role of specific microbiomes in inflammatory processes and interaction of effective anti-inflammatory compounds with this system can not only enhance our understanding of the gut-brain axis, but can also lead to novel intervention in inflammatory diseases.

## Conclusion

Overall, our findings indicate that chronic administration of ketamine results in significant increases in the levels of low-abundance bacteria genera (e.g. *Lactobacillus*, *Turicibacter* and *Sarcina*), and significant decreases in opportunistic pathogens (e.g. *Ruminococcus* and *Mucispirallum*) in male Wistar rats. Thus, it may be suggested that divergent changes in colonic microbiota, where there are increases in probiotic and decreases in pathogenic genera, may in part contribute to the sustained antidepressant and anti-inflammatory effects of ketamine. Clearly, further detailed functional analysis of the role of individual bacterial species and their interactions with central and peripheral mediators of affective behavior or inflammatory processes is warranted. In this vein possible gender effects should also be taken into consideration [83].

## Abbreviations

*AMPA*: α-amino-3-hydroxy-5-methyl-4-isoxazolepropionic acid
*BDNF*: Brain derived neurotrophic factor
*CNS*: Central nervous system
*HDAC*: Histone Deacetylase
*IBS*: Inflammatory bowel syndrome
*IL-1β*: Interleukin-1-beta
*IL-6*: Interleukin-6
*IP*: Intraperitoneally
*LPS*: lipopolysaccharide
*MDD*: Major depressive disorder
*mTOR*: Mammalian target of rapamycin
mTOR: The mammalian target of rapamycin
*NMDA*: N-methyl-d-aspartate
*OTU*: Operational taxonomic unit
*RDP*: Ribosomal Database Project
*SCFAs*: Short chain fatty acids
*TNF-* α: Tumor necrosis factor
